# Prevalence of cardiovascular risk factors by HIV status in a population‐based cohort in South Central Uganda: a cross‐sectional survey

**DOI:** 10.1002/jia2.25901

**Published:** 2022-04-13

**Authors:** Rocio Enriquez, Robert Ssekubugu, Anthony Ndyanabo, Gaetano Marrone, Bruna Gigante, Larry W. Chang, Steven J. Reynolds, Fred Nalugoda, Anna Mia Ekstrom, Nelson K. Sewankambo, David M. Serwadda, Helena Nordenstedt

**Affiliations:** ^1^ Department of Global Public Health Karolinska Institutet Stockholm Sweden; ^2^ Rakai Health Sciences Program Kalisizo Uganda; ^3^ Department of Medicine Karolinska Institutet Stockholm Sweden; ^4^ Department of Epidemiology Johns Hopkins Bloomberg School of Public Health Baltimore Maryland USA; ^5^ Division of Infectious Diseases Department of Medicine Johns Hopkins School of Medicine Baltimore Maryland USA; ^6^ Laboratory of Immunoregulation Division of Intramural Research National Institute for Allergy and Infectious Diseases National Institutes of Health Bethesda Maryland USA; ^7^ Department of Infectious Diseases South Central Hospital Stockholm Sweden; ^8^ Department of Medicine Makerere University School of Medicine Kampala Uganda; ^9^ Department of Disease Control and Environmental Health Makerere University School of Public Health Kampala Uganda; ^10^ Department of Internal Medicine and Infectious Diseases Danderyd University Hospital Stockholm Sweden

**Keywords:** Africa and LMIC, cardiovascular diseases, dyslipidemias, HIV epidemiology, risk factors

## Abstract

**Introduction:**

Cardiovascular disease is one of the leading causes of mortality for people living with HIV, but limited population‐based data are available from sub‐Saharan Africa. This study aimed to determine the prevalence of key cardiovascular disease risk factors, 10‐year risk of cardiovascular disease and type 2 diabetes mellitus through risk scores by HIV status, as well as investigate factors associated with hyperglycaemia, hypertension and dyslipidaemia in South‐Central Uganda.

**Methods:**

A cross‐sectional study was conducted in 37 communities of the population‐based Rakai Community Cohort Study from May 2016 to May 2018. In total, 990 people living with HIV and 978 HIV‐negative participants aged 35–49 years were included. Prevalence estimates and 10‐year cardiovascular and type 2 diabetes risk were calculated by sex and HIV serostatus. Multivariable logistic regression was used to determine associations between socio‐demographic, lifestyle and body composition risk factors and hyperglycaemia, hypertension and dyslipidaemia.

**Results:**

Overweight (21%), obesity (9%), abdominal obesity (15%), hypertension (17%) and low high‐density lipoprotein (HDL) (63%) were the most common cardiovascular risk factors found in our population. These risk factors were found to be less common in people living with HIV apart from hypertension. Ten‐year risk for cardiovascular and type 2 diabetes mellitus risk was low in this population with <1% categorized as high risk. In HIV‐adjusted multivariable analysis, obesity was associated with a higher odds of hypertension (odds ratio [OR] = 2.31, 95% confidence interval [CI] 1.35–3.96) and high triglycerides (OR = 2.08, CI 1.25–3.47), and abdominal obesity was associated with a higher odds of high triglycerides (OR = 2.55, CI 1.55–4.18) and low HDL (OR = 1.36, CI 1.09–1.71). A positive HIV status was associated with a lower odds of low HDL (OR = 0.43, CI 0.35–0.52).

**Conclusions:**

In this population‐based study in Uganda, cardiovascular risk factors of obesity, abdominal obesity, hypertension and dyslipidaemia were found to be common, while hyperglycaemia was less common. Ten‐year risk for cardiovascular and type 2 diabetes mellitus risk was low. The majority of cardiovascular risk factors were not affected by HIV status. The high prevalence of dyslipidaemia in our study requires further research.

## 1 INTRODUCTION

The burden of cardiovascular diseases (CVDs) is on the rise in sub‐Saharan Africa (SSA) due to increasing life expectancy, urbanization and changes in lifestyle and diet across the continent [1]. Across many SSA countries, resource‐limited health systems will not only need to adapt to respond to CVD, but also continue to address the double burden of communicable diseases, childhood illnesses and maternal health as they will remain important causes of morbidity and mortality [2].

The need to adapt health systems in SSA towards CVD prevention and care is further compounded by the HIV epidemic. SSA is home to over 70% of the people living with HIV population and as a positive result of increased access to antiretroviral therapy (ART), life expectancy for this population is increasing [3, 4]. Co‐morbidities, such as CVD, non‐AIDS‐related malignancies and liver disease, already are the leading causes of mortality [5]. Research from high‐income countries has demonstrated that people living with HIV have a 1.5‐2‐fold increased risk for CVD and as they age, both the likelihood of developing and the overall burden of CVD will increase [6, 7, 8]. It is predicted that by 2040, the number of people living with HIV over the age of 50 in SSA will reach 10 million [9]. Consequently, the current health system constraints for the delivery of CVD care in SSA [10, 11] present a situation in which people living with HIV may experience a reduction in quality of life or die, not as a direct result of their HIV infection, but as an indirect result of their increased CVD risk.

Many SSA countries have fragmented health‐information systems that either generate partial or no morbidity and mortality data in relation to CVD, and even less so in relation to HIV [12]. Large‐scale population‐based HIV cohorts can to some extent fill these data gaps and inform health services and care. However, studies that assess CVD burden or risk by HIV status drawn from population‐based cohort studies across the SSA region are scarce, and suggestive that people living with HIV are at an increased burden for dyslipidaemia as compared to the general population [13].

In order to build upon available research findings drawn from HIV focused population‐based cohorts, we aimed to describe the pattern of key CVD risk factors, 10‐year CVD, and type 2 diabetes mellitus (T2DM) risk by HIV status as well as investigate socio‐demographic, lifestyle and body composition risk factors associated with hyperglycaemia, hypertension and dyslipidaemia.

## 2 METHODS

This cross‐sectional study is based on data collected in the 18th survey round of the Rakai Community Cohort Study (RCCS) that occurred from May 2016 to May 2018. The RCCS has been described in detail before [14, 15] but in summary, it is an open population‐based cohort established in 1994 by the Rakai Health Sciences Program (RHSP) in the Rakai region, a predominately rural area in South‐Central Uganda. The catchment area of the RCCS is comprised of 37 non‐fishing communities and four fishing communities. For possible inclusion into the RCCS, participants must be between the ages of 15–49 and be resident for at least 6 months in communities included since inception or at least 1 month with intention to stay longer in the fishing communities.

Participants who meet the enrolment criteria and provide informed consent are then interviewed to assess demographics, sexual and health‐seeking behaviours, and HIV service uptake. Anthropometric measurements, including weight, height and waist circumference, are taken while the participant wears light clothing, and two blood pressure measurements are captured at least 5 minutes apart with the participant seated and with their legs uncrossed. Free HIV testing based on a three rapid test algorithm is provided with results and counselling offered to participants by on‐site counsellors [16]. Finally, non‐fasting venous blood samples are collected, centrifuged in the field using a portable centrifuge, transported the same day to the station office and stored for both planned and future analyses.

### 2.1 Sample selection

**Figure 1:**
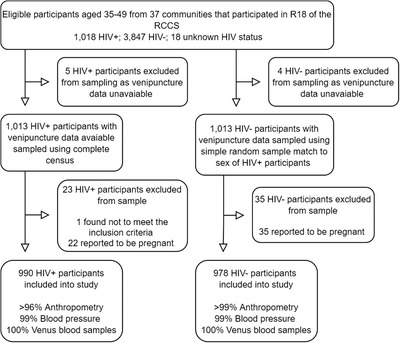
Participant selection for study.

In the 18th study round, there was a total of 4865 participants aged 35–49 with known HIV status (1018 people living with HIVparticipants and 3847 HIV‐negative participants), determined either in 18th survey round or in a previous survey round, who resided in the 37 non‐fishing communities. The sample was focused on participants aged 35–49 as increasing age is predictive of CVD events and are the oldest age group sampled in the cohort [17]. Additionally, participants from the four fishing communities were excluded due to the known high turnover rate of participants in these communities. A total of 1013 participants living with HIV with venous blood data were included in the initial study sample and then matched by sex 1:1 to HIV‐negative participants with venous blood data using simple random sample resulting in a total sample of 2026 participants. The remaining 2834 HIV‐negative participants were not included into the sample due to resource constraints associated with processing the venous blood samples. In total, 1968 participants were included in the final study sample (990 PLHIV) with 58 participants excluded as one was found to not meet the inclusion criteria and 57 reported to be pregnant, which can impact blood pressure and laboratory measurements. Figure 1 details participant selection.

### 2.2 Variable definitions used

The selection of variables for inclusion in this study was drawn from well‐established socio‐demographic, lifestyle‐related and physiological CVD risk factors. Definitions of body composition, blood pressure and relevant blood measures were taken from Ugandan standards [18] (body mass index [BMI], waist circumference, waist‐to‐hip and hypertension) and if no Ugandan standard could be found, definitions were drawn from international standards (estimated glomerular filtration rate [eGFR], hyperglycaemia and dyslipidaemia). BMI was defined as <18.5 kg/m^2^ for underweight, ≥18.5 to <25 kg/m^2^ for normal, ≥25 to <30 kg/m^2^ for overweight and ≥30 kg/m^2^ for obese. For abdominal obesity, a waist circumference of ≥95 to ≤102 cm for males and ≥81 to ≤88 cm for females was defined as “increased risk for CVD” and ≥103 cm for males and ≥89 cm for females as “substantially at increased risk for CVD.” A waist‐to‐hip ratio of ≥0.90 for males and ≥0.85 for females was defined as “substantially at increased risk for CVD.” Hypertension was defined as a systolic blood pressure of ≥140 mmHg, or a diastolic blood pressure of ≥90 mmHg from two blood pressure measurements, or the participant reporting being on hypertension medication. eGFR was calculated using the 2021 Chronic Kidney Disease Epidemiology Collaboration (CKD‐EPI) equation [19] and ≥90 ml/min/1.73 m^2^ was defined as normal/high, ≥60 to ≤ 89 ml/min/1.73 m^2^ as mildly decreased, ≥15 to ≤59 ml/min/1.73 m^2^ as moderately to severely decreased and <15 ml/min/1.73 m^2^ as kidney failure. Hyperglycaemia was defined as a random plasma glucose ≥7.8 mmol/l or participant reporting being on antidiabetic medication [20, 21]. Dyslipidaemia was defined as either any of the following conditions: high total cholesterol >5.0 mmol/l; [22] low‐density lipoprotein (LDL) cholesterol >3.0 mmol/l; [23] high‐density lipoprotein (HDL) cholesterol as <1.03 mmol/l for men and <1.29 mmol/l for women; [24] and finally high triglycerides ≥1.7 mmol/l [24].

Socio‐demographic variables included sex, age, residence location (categorized as rural and semi‐urban), education attainment (none, <5 years and ≥5 years), occupation (agricultural and non‐agricultural focused), religion (none, Catholic, Protestant and Muslim) and marital status (single and married). Lifestyle‐related CVD variables included alcohol consumption (none, infrequent defined as last drink more than a month ago and frequent defined as last drink less than a month ago), smoking status (smoker or non‐smoker), duration of smoking (1–10 years, 11–20 and 21 or more years), physical activity (exercising above or below 30 minutes per day) and fruit and vegetable consumption (consuming either above or below the daily median consumption of fruit and vegetables among the surveyed participants).

Ten‐year risk of CVD and T2DM risk scores were calculated for each participant. For 10‐year CVD risk, both non‐laboratory‐based and laboratory‐based Framingham risk scores were calculated [25]. The non‐laboratory‐based model, which requires only history and physical examination measurements, included sex, age, systolic blood pressure, current treatment for hypertension, smoking status, diabetes and BMI. The laboratory‐based model used the same risk factors but replaced BMI with total cholesterol and HDL. Ten‐year T2DM risk was calculated using a modified version of the FINDRISC model [26]. The FINDRISC model includes age, BMI, waist circumference, hypertension medication, history of high blood glucose, family history of diabetes, daily fruit and vegetable consumption, and physical activity. The adapted FINDRISC risk score model included all these variables except for information on history of diabetes in extended family members, which resulted in a possible total of 35 points as compared to 38 in the standard model.

### 2.3 Statistical analyses

Baseline characteristics and prevalence rates of CVD risk factors, 10‐year CVD and T2DM risk were tabulated by sex and HIV status with differences assessed by either two tailed *t*‐test, chi‐squared test or Fisher's exact test, respectively. Among participants living with HIV, a secondary analysis was conducted to assess the differences by ART use (use or no use) for hyperglycaemia, hypertension, triglycerides and HDL by chi‐squared test. The agreement between the two Framingham CVD risk score models, non‐lab‐based and lab‐based, was assessed using kappa statistics. Adjusted by HIV status, multivariable logistic regression was used to identify variables associated with hyperglycaemia, hypertension and dyslipidaemia, specifically high triglycerides and low HDL given that they were found to have the highest prevalence estimates in our sample. Variables explored included socio‐demographic factors (age, residence location, occupation and education), CVD‐related lifestyle (smoking, physical activity and daily fruit and vegetable consumption) and body composition (BMI, waist circumference and waist‐to‐hip ratio). Alcohol was excluded from the analysis as quantities of alcohol were not assessed during data collection. Variables that had a *p*‐value of 0.20 in bivariate analysis were included in the HIV‐adjusted multivariable analysis with variables that had a *p*‐value of <0.05 considered to be statistically significant. Stata 15 (Stata Corporation, College Station, TX, USA) was used for all analyses.

### 2.4 Ethics

The study was approved by the Uganda Virus Research Institute Research and Ethics Committee; Protocol GC/127/18/07/657, and Swedish Ethical Review Authority (2018/2542‐31/2) and the Uganda National Council for Science and Technology for clearance (HS540). All participants provided written informed consent as part of the RCCS activities and were compensated for their time.

## 3 RESULTS

### 3.1 CVD risk factors in study participants

Complete socio‐demographic and lifestyle‐related CVD risk factors by sex and HIV status are presented in Table 1 for the sampled population. Full cohort characteristics, including those for which venous blood samples were not processed, are given in online Appendix Table A. For socio‐demographic variables, differences in residence location, education attainment and marital status were found. Female participants living with HIV were more likely to report living in semi‐urban areas as compared to female HIV‐negative participants (51% vs. 45%, *p* = 0.03). Fewer participants living with HIV reported the highest education attainment as compared to HIV‐negative participants (69% vs. 74%, *p* = 0.03) with a greater difference reported among females (67% vs. 75%, *p*<0.01). Furthermore, participants living with HIV reported lower marriage rates as compared to HIV‐negative participants (55% vs. 72%, *p*<0.001). The prevalence of reported lifestyle‐related CVD risk factors did not differ substantially by HIV status apart from participants living with HIV reporting higher consumption levels of fruit and vegetables (58% vs. 51%, *p*<0.001) as compared to HIV‐negative participants.

**Table 1:** Participant characteristics by sex and HIV status

	Females		Males		Total	
Characteristic	HIV+ No. (%)	HIV– No. (%)	HIV+ No. (%)	HIV– No. (%)	HIV+ No. (%)	HIV– No. (%)
Age	*n* = 630	*n* = 619	*n* = 360	*n* = 359	*n* = 990	*n* = 978
Age, mean (SD in years)	41 (4)	41 (4)	41 (4)	41 (4)	41 (4)	41 (4)
35–39	252 (40%)	270 (44%)	135 (38%)	136 (38%)	387 (39%)	406 (42%)
40–44	234 (37%)	224 (36%)	128 (36%)	124 (35%)	362 (37%)	348 (36%)
45–49	144 (23%)	125 (20%)	97 (27%)	99 (28%)	241 (24%)	224 (23%)
Residence location	*n* = 630	*n* = 619	*n* = 360	*n* = 359	*n* = 990	*n* = 978
Rural	310 (49%)	342 (55%)	196 (54%)	195 (54%)	506 (51%)	537 (55%)
Semi‐urban	320 (51%)	277 (45%)	164 (46%)	164 (46%)	484 (49%)	441 (45%)
Education	*n* = 630	*n* = 618	*n* = 360	*n* = 359	*n* = 990	*n* = 978
No education	49 (8%)	35 (6%)	17 (5%)	17 (5%)	66 (7%)	52 (5%)
<5 Years of study	160 (25%)	119 (19%)	83 (23%)	80 (22%)	243 (25%)	199 (20%)
≥5 Years of study	421 (67%)	464 (75%)	260 (72%)	262 (73%)	681 (69%)	726 (74%)
Occupation^a^	*n* = 630	*n* = 619	*n* = 360	*n* = 359	*n* = 990	*n* = 978
Agricultural focused	379 (60%)	392 (63%)	166 (46%)	152 (42%)	545 (55%)	544 (56%)
Non‐agricultural focused	251 (40%)	227 (37%)	192 (53%)	207 (58%)	443 (45%)	434 (44%)
Marital status	*n* = 630	*n* = 619	*n* = 360	*n* = 359	*n* = 990	*n* = 978
Single	353 (56%)	196 (32%)	96 (27%)	73 (20%)	449 (45%)	269 (28%)
Married	277 (44%)	423 (69%)	264 (73%)	286 (80%)	541 (55%)	709 (72%)
Religion	*n* = 630	*n* = 619	*n* = 360	*n* = 359	*n* = 990	*n* = 978
None	2 (<1%)	6 (<1%)	0 (0%)	2 (<1%)	2 (<1%)	8 (<1%)
Catholic	429 (68%)	410 (66%)	258 (72%)	234 (65%)	687 (69%)	644 (66%)
Protestant	138 (22%)	122 (20%)	73 (20%)	76 (21%)	211 (21%)	198 (20%)
Muslim	61 (10%)	81 (13%)	29 (8%)	47 (13%)	90 (9%)	128 (13%)
Alcohol consumption	*n* = 628	*n* = 619	*n* = 360	*n* = 358	*n* = 988	*n* = 977
No	400 (64%)	363 (59%)	130 (36%)	118 (33%)	530 (54%)	481 (49%)
Infrequent (last drink >1 month)	39 (6%)	57 (9%)	28 (8%)	20 (6%)	67 (7%)	77 (8%)
Frequent (last drink ≤1 month)	189 (30%)	199 (32%)	202 (56%)	220 (61%)	391 (40%)	419 (43%)
Smoking status	*n* = 630	*n* = 619	*n* = 360	*n* = 359	*n* = 990	*n* = 978
Non‐smoker	613 (97%)	591 (96%)	292 (81%)	290 (81%)	905 (91%)	881 (90%)
Smoker	17 (3%)	28 (5%)	68 (19%)	69 (19%)	85 (9%)	97 (10%)
Duration of smoking	*n* = 17	*n* = 28	*n* = 68	*n* = 69	*n* = 85	*n* = 97
1–10 Years	10 (59%)	23 (82%)	34 (50%)	38 (55%)	44 (52%)	61 (63%)
11–20 Years	6 (35%)	5 (18%)	25 (37%)	18 (26%)	31 (36%)	23 (24%)
21+ Years	1 (6%)	0 (0%)	9 (13%)	13 (19%)	10 (12%)	13 (13%)
Physical activity	*n* = 629	*n* = 618	*n* = 359	*n* = 358	*n* = 988	*n* = 976
Physically active (>30 minutes)	609 (97%)	603 (98%)	342 (95%)	345 (96%)	951 (96%)	948 (97%)
Physically inactive (≤30 minutes)	20 (3%)	15 (2%)	17 (5%)	13 (4%)	37 (4%)	28 (3%)
Daily fruit and vegetable consumption	*n* = 630	*n* = 619	*n* = 360	*n* = 359	*n* = 990	*n* = 978
Consumption, median servings	1.4	1.1	1	0.9	1.3	1.1
Below average (<1.14 servings)	209 (33%)	264 (43%)	202 (56%)	218 (61%)	411 (42%)	482 (49%)
Above average (≥1.14 servings)	421 (67%)	355 (58%)	158 (44%)	141 (39%)	579 (58%)	496 (51%)

Abbreviation: SD, standard deviation.

^a^ Information on those who reported to be unemployed (two HIV+ males) not included in the table but included in the denominator shown.

**Figure 2:**
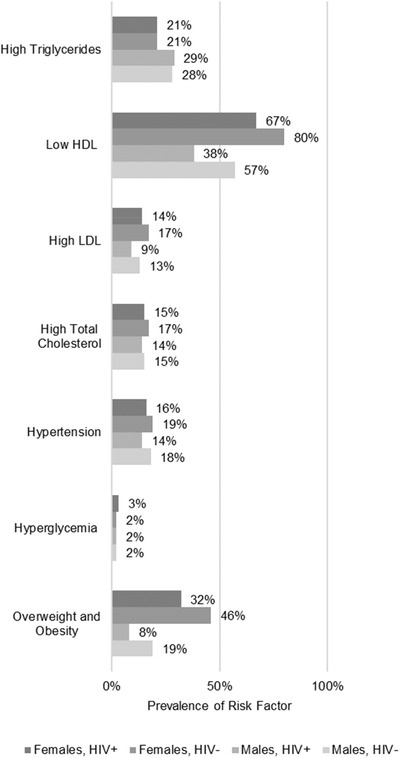
Prevalence of CVD risk factors by population of interest: (a) females, HIV+, (b) females, HIV–, (c) males, HIV+ and (d) males HIV–.

**Figure 3:**
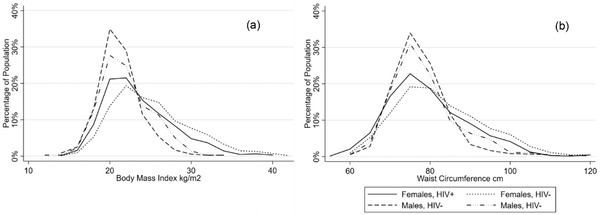
Distribution of BMI (a) and waist circumference (b) by sex and HIV status.

**Figure 4:**
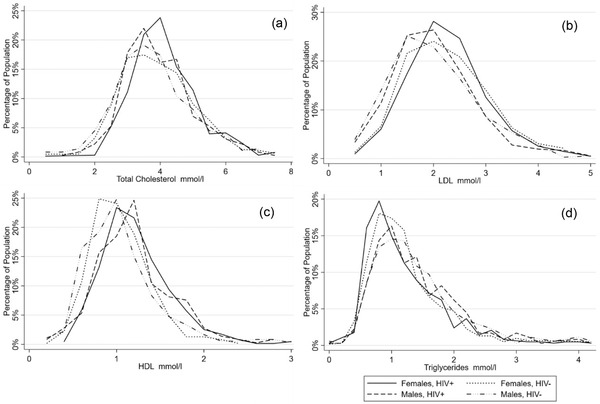
Distribution of lipid profiles by sex and HIV status.

In our population, several differences in anthropometric measurements of BMI, waist circumference and waist‐to‐hip ratio were found (Table 2 and Figure 2). Participants living with HIV had lower levels of obesity and abdominal obesity as compared to the HIV‐negative participants (6% vs. 12%, *p*<0.001 and 12% vs. 18%, *p*<0.001, respectively). Regardless of HIV status, female participants were found to have higher rates of being overweight, obesity and abdominal obesity as compared to males. Finally, male participants living with HIV were found to have high waist‐to‐hip ratio measurement as compared to male HIV‐negative participants (31% vs. 22%, *p* = 0.005). Only minimal differences to blood pressure and laboratory measurements were found by HIV status in our population apart from low HDL. Participants living with HIV were less likely to be found with low HDL as compared to HIV‐negative participants (54% vs. 72%, *p*<0.001), with higher levels of low HDL found in females as compared to males. Figures 3 and 4 present the distribution of BMI, waist circumference and lipid profiles by sex and HIV status.

**Table 2:** Prevalence of cardiovascular risk factors by sex and HIV status

	Females		Males		Total		
Risk factor	HIV+No. (%)	HIV–No. (%)	HIV+No. (%)	HIV–No. (%)	HIV+No. (%)	HIV–No. (%)	*p*‐Value^a^
Anthropometric measurements							
BMI	*n* = 629	*n* = 619	*n* = 357	*n* = 357	*n* = 986	*n* = 976	<0.001
Underweight (<18.5 kg/m^2^)	44 (7%)	28 (5%)	38 (11%)	37 (10%)	82 (8%)	65 (7%)	
Normal (≥18.5 to <25 kg/m^2^)	384 (61%)	309 (50%)	289 (81%)	253 (71%)	673 (68%)	562 (58%)	
Overweight (≥25 to <30 kg/m^2^)	145 (23%)	170 (27%)	27 (8%)	62 (17%)	172 (17%)	232 (24%)	
Obese (≥30 kg/m^2^)	56 (9%)	112 (18%)	3 (<1%)	5 (1%)	59 (6%)	117 (12%)	
Waist circumference	*n* = 629	*n* = 615	*n* = 359	*n* = 358	*n* = 988	*n* = 973	<0.001
Not at risk (males: <95 cm and females: <81 cm)	365 (58%)	295 (48%)	352 (98%)	338 (94%)	717 (73%)	633 (65%)	
Increased risk (males: ≥95 to ≤102 cm and females: ≥81 to ≤88 cm)	145 (23%)	149 (24%)	5 (1%)	14 (4%)	150 (15%)	163 (17%)	
Substantially increased risk (males: ≥103 cm and females: ≥89 cm)	119 (19%)	171 (28%)	2 (1%)	6 (2%)	121 (12%)	177 (18%)	
Waist‐to‐hip ratio	*n* = 629	*n* = 614	*n* = 359	*n* = 357	*n* = 988	*n* = 971	0.36
Not at risk (males: <0.90 and females: <0.85)	330 (52%)	308 (50%)	247 (69%)	279 (78%)	577 (58%)	587 (60%)	
Substantially increased risk (males: ≥0.90 and females: ≥0.85)	299 (48%)	306 (50%)	112 (31%)	78 (22%)	411 (42%)	384 (40%)	
Blood pressure measurements							
Hypertension^b^	*n* = 614	*n* = 622	*n* = 355	*n* = 356	*n* = 977	*n* = 970	0.06
Normal (<120/80 mmHg)	294 (47%)	265 (43%)	156 (44%)	134 (38%)	450 (46%)	399 (41%)	
Pre‐hypertension (≥120/80 mmHg to <140/90 mmHg)	226 (36%)	233 (38%)	149 (42%)	158 (44%)	375 (38%)	391 (40%)	
Hypertension (≥140/90 mmHg or on medication)	102 (16%)	116 (19%)	50 (14%)	64 (18%)	152 (16%)	180 (19%)	
Laboratory measurements							
Random plasma glucose^c^	*n* = 630	*n* = 619	*n* = 360	*n* = 359	*n* = 990	*n* = 978	0.67
Normal (<7.8 mmol/l)	613 (97%)	605 (98%)	354 (98%)	353 (98%)	967 (98%)	958 (98%)	
Hyperglycaemia (≥7.8 mmol/l or on medication)	17 (3%)	14 (2%)	6 (2%)	6 (2%)	23 (2%)	20 (2%)	
Diabetes (>11.1 mmol/l or on medication)	9 (1%)	8 (1%)	1 (<1%)	1 (<1%)	10 (<1%)	9 (<1%)	0.84
Kidney function	*n* = 630	*n* = 619	*n* = 360	*n* = 359	*n* = 990	*n* = 978	
Creatinine, mean (μmol/l)	65.78	63.70	76.21	76.03	69.57	68.22	0.07
Estimated glomerular filtration rate^d^							0.10
Normal (≥90 ml/min/1.73 m^2^)	480 (76%)	508 (82%)	327 (91%)	324 (90%)	807 (82%)	832 (85%)	
Mildly decreased (≤89 to ≥60 ml/min/1.73 m^2^)	145 (23%)	107 (17%)	32 (9%)	33 (9%)	177 (18%)	140 (14%)	
Moderately severely decreased (≤ 59 to ≥15 ml/min/1.73 m^2^)	5 (<1%)	4 (<1%)	1 (<1%)	2 (<1%)	26(<1%)	6 (<1%)	
Lipid profile	*n* = 630	*n* = 619	*n* = 360	*n* = 359	*n* = 990	*n* = 978	
Total cholesterol							0.66
Normal (≤5 mmol/l)	533 (85%)	518 (84%)	308 (86%)	306 (85%)	872 (88%)	855 (87%)	
High (>5 mmol/l)	97 (15%)	101 (16%)	52 (14%)	53 (15%)	118 (12%)	123 (13%)	
LDL							0.40
Normal (≤3 mmol/l)	540 (86%)	514 (83%)	327 (91%)	311 (87%)	867 (88%)	825 (84%)	
High (>3 mmol/l)	90 (14%)	105 (17%)	33 (9%)	48 (13%)	123 (12%)	153 (16%)	
HDL							<0.001
Normal (≥1.03 mmol/l in males and ≥1.29 mmol/l in females)	228 (36%)	120 (19%)	225 (63%)	155 (43%)	453 (46%)	275 (28%)	
Low (males: <1.03 mmol/l and females: <1.29 mmol/l)	402 (64%)	499 (81%)	135 (38%)	204 (57%)	537 (54%)	703 (72%)	
Triglycerides							0.47
Normal (<1.7 mmol/l)	504 (80%)	504 (81%)	256 (71%)	260 (72%)	760 (77%)	764 (78%)	
High (≥1.7 mmol/l)	126 (20%)	115 (19%)	104 (29%)	99 (28%)	230 (23%)	214 (22%)	

Abbreviations: BMI, body mass index; HDL, high‐density lipoprotein; LDL, low‐density lipoprotein.

^a^ *p*‐Values presented in table are either calculated using chi‐squared test, *t*‐test or Fisher's exact test and only calculated to assess the difference by HIV status.

^b^ Includes those who were found to have hypertension or reported taking medication for hypertension. All 51 who reported to be taking hypertension medication were also found to have hypertension based on blood pressure medication.

^c^ Includes 12 participants reported to be on medication. The denominator used under diabetes is that of the entire population and not those who were found to have hyperglycaemia.

^d^ Information on kidney failure (<15 ml/min/1.73 m^2^) category not presented as no cases fell into this category.

### 3.2 Ten‐year CVD and T2DM risk by Framingham and adapted FINDRISC scores

CVD and T2DM risk scores by sex and HIV status are presented in Table 3. Overall, 10‐year CVD risk was found to be low in our population, with either the non‐lab‐based or lab‐based Framingham CVD risk score models. Less than 1% of participants were categorized as high risk in both models. Between the two Framingham CVD risk models, an interrater agreement of 97.5% was observed (kappa 0.56, *p*<0.001). For the adapted FINDRISC risk score, less than 1% of participants were categorized as high risk and 0% of participants were categorized as very high risk for developing T2DM in the next 10 years with no differences found by HIV status.

**Table 3:** Risk scores by sex and HIV status

	Females		Males		Total		
	HIV+ No. (%)	HIV– No. (%)	HIV+ No. (%)	HIV– No. (%)	HIV+ No. (%)	HIV– No. (%)	*p*‐Value^a^
Non‐Lab Framingham^b^	*n* = 621	*n* = 613	*n* = 351	*n* = 354	*n* = 972	*n* = 967	0.46
Low risk (<10)	612 (99%)	607 (99%)	336 (96%)	327 (93%)	948 (98%)	934 (97%)	
Intermediate risk (10–20)	8 (1%)	6 (1%)	15 (4%)	26 (7%)	23 (2%)	32 (3%)	
High risk (>20)	1 (<1%)	0 (0%)	0 (0%)	1 (<1%)	1 (<1%)	1 (<1%)	
Lab Framingham^c^	*n* = 622	*n* = 613	*n* = 354	*n* = 356	*n* = 976	*n* = 969	0.17
Low risk (<10)	611 (99%)	604 (99%)	344 (97%)	325 (93%)	955 (98%)	935 (96%)	
Intermediate risk (10–20)	10 (2%)	9 (1%)	10 (3%)	22 (6%)	20 (2%)	31 (3%)	
High risk (>20)	1 (<1%)	0 (0%)	0 (0%)	3 (1%)	1 (<1%)	3 (<1%)	
Adapted FINDRISC^d^	*n* = 628	*n* = 613	*n* = 356	*n* = 356	*n* = 984	*n* = 969	0.06
Low (1 in 100)	501 (80%)	458 (75%)	343 (96%)	329 (92%)	844 (86%)	787 (81%)	
Slightly elevated (1 in 25)	104 (17%)	122 (20%)	11 (3%)	25 (7%)	115 (12%)	147 (15%)	
Moderate (1 in 6)	17 (3%)	26 (4%)	2 (<1%)	2 (<1%)	19 (2%)	28 (3%)	
High (1 in 3)	6 (1%)	7 (1%)	0 (0%)	0 (0%)	6 (<1%)	7 (<1%)	
Very high (1 in 2)	0 (0%)	0 (0%)	0 (0%)	0 (0%)	0 (0%)	0 (0%)	

Abbreviation: FINDRISC, Finnish Diabetes Risk Score.

^a^ *p*‐Values only calculated to assess the difference by HIV status.

^b^ Non‐lab Framingham Scores were calculated using data on sex, age, systolic blood pressure, current treatment for hypertension smoking status, diabetes and BMI. Final risk score indicates the likelihood of developing coronary heart disease in the next 10 years.

^c^ Lab‐based Framingham Scores were calculated using data on sex, age, systolic blood pressure, current treatment for hypertension smoking status, diabetes, total cholesterol and HDL. Final risk score indicates the likelihood of developing coronary heart disease in the next 10 years.

^d^ Final score calculation excludes three pts as the question inquiring whether extending family members have diabetes was not included in the study questionnaire. Therefore, the total number of possible points equalled to 35 instead of 38 in the standard FINDRISC score calculator.

^e^ One in 100 will on average develop diabetes in the next 10 years.

### 3.3 Associated risk factors with hyperglycaemia, hypertension and dyslipidaemia

Factors associated with hyperglycaemia, hypertension and dyslipidaemia (high triglycerides and low HDL) by HIV‐adjusted multivariate logistic regression analysis are presented in Table 4. Factors associated with total cholesterol and high LDL for dyslipidaemia are given in online Appendix Table B. Older ages of 40–44 (odds ratio [OR] = 1.57, 95% confidence interval [CI] 1.18–2.10) and 45–49 (OR = 2.04, CI 1.49–2.80), living in a semi‐urban area (OR = 1.49, CI 1.16–1.90), being overweight (OR = 2.27, CI 1.62–3.20) and being obese (OR = 2.31, CI 1.35–3.96) were associated with a higher odds of hypertension. Being male (OR = 3.30, CI 2.46–4.42), older age of 45–49 (OR = 1.36, CI 1.03–1.81), being obese (OR = 2.08, CI 1.25–3.47), being at either increased or substantially at increased risk for CVD from waist circumference measurements ([OR = 1.57, CI 1.06–2.32] and [OR = 2.55, CI 1.55–4.18], respectively) and substantially at increased risk for CVD from waist‐to‐hip ratio measurements (OR = 1.93, CI 1.50–2.49) were associated with a higher odds of high triglycerides. Being substantially at increased risk for CVD from waist‐to‐hip ratio measurements (OR = 1.36, CI 1.09–1.71) was associated with higher odds of low HDL, while being HIV positive (OR = 0.43, CI 0.35–0.52), male (OR = 0.42, CI 0.33–0.53) and being underweight (OR = 0.64, CI 0.44–0.92) were associated with a lower odds of low HDL. No associations between HIV status, sex, age, residence location, education, occupation, physical activity, daily fruit and vegetable consumption, BMI, waist circumference or waist‐to‐hip ratio with hyperglycaemia were found.

**Table 4:** Factors associated with hyperglycaemia, hypertension, high triglycerides and low HDL

	Hyperglycaemia		Hypertension	
	Univariable	Multivariable	Univariable	Multivariable
	OR (95% CI)	OR (95% CI)	OR (95% CI)	OR (95% CI)
HIV status				
HIV–	Ref.	Ref.	**Ref**.	Ref.
HIV+	1.14 (0.62–2.09)	1.19 (0.64–2.19)	**0.81 (0.64–1.02)**	0.87 (0.68–1.12)
Sex				
Female	Ref.	–	Ref.	–
Male	0.67 (0.34–1.31)	–	0.89 (0.70–1.14)	–
Age				
35–39	Ref.	–	**Ref**.	**Ref**.
40–44	0.85 (0.41–1.77)	–	**1.45 (1.10–1.93)**	**1.57 (1.18–2.10)** ***
45–49	1.31 (0.63–2.73)	–	**1.81 (1.34–2.45)**	**2.04 (1.49–2.80)** ****
Residence location				
Rural	Ref.	–	**Ref**.	Ref.
Semi‐urban	1.19 (0.65–2.17)	–	**1.58 (1.25–2.01)**	**1.49 (1.16–1.90)** ***
Education				
No education	0.85 (0.20–3.61)	–	0.63 (0.35–1.12)	–
<5 years of study	1.49 (0.77–2.91)	–	0.94 (0.71–1.25)	–
≥5 years of study	Ref.	–	Ref.	–
Occupation				
Agricultural focused	**0.72 (0.53–0.98)**	0.74 (0.54–1.01)	1.00 (0.90–1.14)	–
Non‐agricultural focused	**Ref**.	Ref.	Ref.	–
Smoking status				
Non‐smoker	Ref.	–	**Ref**.	Ref.
Current smoker	1.01 (0.36–2.85)	–	0.48 (0.29–0.80)**	0.59 (0.35–1.10)
Physical activity				
Physically active (>30 minutes)	Ref.	–	Ref.	–
Physically inactive (≤30 minutes)	0.69 (0.09–5.10)	–	1.15 (0.61–2.18)	–
Daily fruit and vegetable consumption				
Below average (<1.14 servings)	0.78 (0.42–1.45)	–	1.14 (0.90–1.44)	–
Above average (≥1.14 servings)	Ref.	–	Ref.	–
BMI				
Underweight (<18.5 kg/m^2^)	0.33 (0.04–2.46)	–	**0.85 (0.49–1.47)**	0.85 (0.49–1.48)
Normal (≥18.5 to <25 kg/m^2^)	Ref.	–	**Ref**.	Ref.
Overweight (≥25 to <30 kg/m^2^)	1.23 (0.58–2.58)	–	**2.56 (1.94–3.38)**	**2.27 (1.62–3.20)** ****
Obese (≥30 kg/m^2^)	2.00 (0.85–4.71)	–	**2.87 (1.99–4.15)**	**2.31 (1.35–3.96)** ***
Waist circumference				
Not at risk (males: <95 cm and females: <81 cm)	**Ref**.	Ref.	**Ref**.	Ref.
Increased risk (males: ≥95 to ≤102 cm and females: ≥81 to ≤88 cm)	**1.79 (0.81–3.92)**	1.58 (0.69–3.63)	**1.45 (1.05–2.01)**	0.95 (0.65–1.37)
Substantially increased risk (males: ≥103 cm and females: ≥89 cm)	**2.53 (1.24–5.18)**	1.99 (0.88–4.51)	**2.56 (1.90–3.43)**	1.14 (0.71–1.84)
Waist‐to‐hip ratio				
Not at risk (males: <0.90 and females: <0.85)	**Ref**.	Ref.	**Ref**.	Ref.
Substantially increased risk (males: ≥0.90 and females: ≥0.85)	**1.88 (1.02–3.45)**	1.41 (0.70–2.84)	**1.40 (1.11–1.78)**	1.05 (0.79–1.38)
HIV status				
HIV–	Ref.	Ref.	**Ref**.	Ref.
HIV+	1.08 (0.87–1.33)	1.21 (0.96–1.51)	**0.46 (0.38–0.56)**	**0.43 (0.35–0.52)** ****
Sex				
Female	**Ref**.	Ref.	**Ref**.	Ref.
Male	**1.65 (1.33–2.04)**	**3.30 (2.46–4.42)** ****	**0.34 (0.28–0.42)**	**0.42 (0.33–0.53)** ****
Age				
35–39	**Ref**.	Ref.	Ref.	–
40–44	**1.10 (0.86–1.41)**	1.07 (0.82–1.38)	0.96 (0.78–1.18)	–
45–49	**1.45 (1.11–1.89)**	**1.36 (1.03–1.81)** **	0.92 (0.73–1.17)	–
Residence location				
Rural	Ref.	–	Ref.	–
Semi‐urban	1.10 (0.89–1.36)	–	0.90 (0.75–1.08)	–
Education				
No education	0.88 (0.56–1.39)	–	**1.73 (1.13–2.65)**	**1.87 (1.19–2.94)** ***
<5 years of study	0.84 (0.64–1.09)	–	**1.09 (0.87–1.36)**	1.21 (0.95–1.53)
≥5 years of study	Ref.	–	Ref.	Ref.
Occupation				
Agricultural focused	**0.85 (0.77–0.95)**	0.97 (0.86–1.09)	**1.19 (1.09–1.31)**	**1.14 (1.03–1.26)** **
Non‐agricultural focused	**Ref**.	Ref.	**Ref**.	**Ref**.
Smoking status				
Non‐smoker	Ref.	–	**Ref**.	Ref.
Current smoker	1.14 (0.80–1.63)	–	**0.52 (0.38–0.70)**	0.76 (0.54–1.07)
Physical activity				
Physically active (>30 minutes)	Ref.	–	Ref.	–
Physically inactive (≤30 minutes)	1.03 (0.57–1.85)	–	0.82 (0.50–1.36)	–
Daily fruit and vegetable consumption				
Below average (<1.14 servings)	1.07 (0.86–1.32)	–	**0.82 (0.68–0.98)**	0.91 (0.75–1.12)
Above average (≥1.14 servings)	Ref.	–	**Ref**.	Ref.
BMI				
Underweight (<18.5 kg/m^2^)	**1.43 (0.95–2.15)**	1.46 (0.96–2.24)	**0.62 (0.44–0.88)**	**0.64 (0.44–0.92)** **
Normal (≥18.5 to <25 kg/m^2^)	**Ref**.	Ref.	**Ref**.	Ref.
Overweight (≥25 to <30 kg/m^2^)	**1.83 (1.41–2.37)**	1.45 (1.04–2.03)	**1.83 (1.43–2.34)**	1.31 (0.96–1.77)
Obese (≥30 kg/m^2^)	**3.18 (2.27–4.44)**	**2.08 (1.25–3.47)** ***	**2.49 (1.71–3.62)**	1.13 (0.66–1.96)
Waist circumference				
Not at risk (males: <95 cm and females: <81 cm)	**Ref**.	Ref.	**Ref**.	Ref.
Increased risk (males: ≥95 to ≤102 cm and females: ≥81 to ≤88 cm)	**1.21 (0.90–1.63)**	**1.57 (1.06–2.32)** **	**1.66 (1.27–2.16)**	0.83 (0.59–1.15)
Substantially increased risk (males: ≥103 cm and females: ≥89 cm)	**2.80 (2.14–3.67)**	**2.55 (1.55–4.18)** ****	**2.91 (2.15–3.94)**	1.28 (0.78–2.09)
Waist‐to‐hip ratio				
Not at risk (males: <0.90 and females: <0.85)	**Ref**.	Ref.	**Ref**.	Ref.
Substantially increased risk (males: ≥0.90 and females: ≥0.85)	**2.23** **(1.80–2.76)**	**1.93 (1.50–2.49)** ****	**1.69 (1.40–2.05)**	**1.36 (1.09–1.71)** ****

Variables that were found to be significant, <.20 in univariable and <.05 in multivariable, are marked in bold. Abbreviation: BMI, body mass index.

Significance *p*‐levels:

** *p* <0.05.

*** *p* <0.01.

**** *p* <0.001.

### 3.4 Secondary analysis on selected CVD outcomes by ART status

Current ART use was reported by 87% of participants living with HIV. Current ART use was associated with a lower prevalence for low HDL and high triglycerides as compared to those who reported no current ART use (52% vs. 72%, *p*<0.001 and 22% vs. 32%, *p* = 0.01, respectively).

## 4 DISCUSSION

We estimated the burden of several CVD risk factors by HIV status among participants aged 35–49 from a large population‐based cohort in South‐Central Uganda. Dyslipidaemia, specifically low HDL, was found to be the most common CVD risk factor. Participants with HIV were found to have a comparable CVD risk factor profile to HIV‐negative participants apart from lower prevalence rates for obesity, abdominal obesity and low HDL. In multivariable logistic regression, a positive HIV status was found to be associated with a lower odds of low HDL.

We found a low prevalence of several key CVD risk factors, and 10‐year CVD risk and T2DM risk scores in our study population with no or small differences by HIV status. Overall, prevalence estimates were consistent with previous published findings from Uganda for smoking (8%) [27], inadequate physical activity (6%) [28] and hyperglycaemia (3% and 2%) [29, 30], and lower for 10‐year CVD risk (9% for BMI‐based formula) [31] drawn from large population‐based cross‐sectional surveys. No previously published estimates could be found for FINDRISC scores in Uganda. In addition to lower rates of obesity, people living with HIV reported less smoking, greater physical activity and higher fruit and vegetable consumption as compared to HIV‐negative participants. A lower CVD risk factor profile among participants living with HIV as compared to HIV‐negative participants has been reported elsewhere in Uganda and South Africa with differences potentially a result of an increased access to routine healthcare and associated CVD preventative counselling for people living with HIV as compared to the general population [32, 33]. Further research is needed to determine the exact reasons for the difference in CVD risk profiles and particularly the role that access to routine healthcare may play in the difference.

A fair agreement between the two Framingham CVD risk scores was found in our population. As lipid profile screening is too costly for many low‐income countries across the SSA region, the validation of the non‐lab‐based Framingham risk score for the region should be explored. However, there are some important caveats to mention. The non‐lab‐based Framingham CVD risk score has been found to correlate well with subclinical atherosclerosis [34], but research comparing its correlation to CVD outcomes is hindered by the lack of available CVD outcome data in the region. Furthermore, in a study from South Africa, the researcher found that overweight and mild obesity was protective against all‐cause mortality, particularly for women [35]. This highlights the importance of understanding region variations to known CVD risk factors and the identification of possible region‐specific CVD risk factors. Although research on the use of the FINDRISC on SSA populations is limited, a study conducted in Botswana found the screening tool only modestly effective in predicting undiagnosed diabetic patients [36]. Finally, anthropometric measurements could offer another potential avenue for prevention and screening of hypertension and dyslipidaemia in rural and resource‐constrained settings. Both general and abdominal obesities were found to be common risk factors in our population from a rural setting and were found to be associated with hypertension and dyslipidaemia.

The high prevalence of dyslipidaemia found in our population, especially of low HDL, is consistent with previous studies conducted across SSA (37%) [37] and studies focused on rural populations in Uganda (71% and 32%, respectively) [27, 31]. Low HDL has been well‐established as an independent risk factor for CVD events in both North American and European populations [38, 39], but the clinical importance of this abnormality for rural sub‐Saharan African settings remains unclear. This is illustrated by the finding that in our population, despite a high prevalence of dyslipidaemia, low 10‐year CVD risk was found as determined by the Framingham CVD risk score. Further studies to determine the causes and clinical importance of low HDL and potentially the development of region‐specific screening thresholds are needed.

Finally, a positive HIV status was found to be negatively associated with low HDL in our population. In a secondary analysis, we found current ART use to be associated with a lower prevalence of low HDL and high triglycerides as compared to those who reported no current ART use. HIV infection is known to promote an early increase of triglycerides and lower levels of HDL, and an eventual decrease in LDL, and ART is known to initially promote higher levels of HDL that then decline over time [39, 40, 41, 42]. Specifically, a 2013 Tanzania study found that after 6 months of initiating ART, HDL increased and then levelled off over a duration of 3 years [43]. Our results support that ART use is associated with increased HDL levels. No associations between HIV infection to either high triglycerides or LDL levels were found in our population. Research focused on understanding the contribution of HIV infection and ART to dyslipidaemia in the SSA context is needed to clarify, if any, changes needed in the delivery of HIV treatment care.

The main strength of this study is the large sample size, and that the sample was drawn from a well‐established population‐based cohort that included almost all participants living with HIV in our age group, and with the comparison population of HIV‐negative participants drawn randomly from the same study population, reducing the risk of selection bias. Few studies across SSA draw their samples in this manner. However, our study has some limitations that are important to mention. Self‐reported data were used for all socio‐economic and CVD lifestyle‐related data presented, which may be subject to social desirability and other biases. Information on type, duration or previous use of ART use was not collected and, therefore, its potential impact on lipid profiles cannot be clarified. Fasting blood samples could not be obtained for glucose or lipid measurements due to data collection procedures of the RCCS in which a participant may be seen over several hours throughout the day making fasting samples impractical for collection. However, the impact of non‐fasting on lipid profiles, including for triglycerides, has been shown to be minimal and in recent years, non‐fasting lipids have been suggested to possibly be even better for CVD risk prediction [44, 45]. Data on extended family (grandparents, uncles or first cousins) were lacking for the calculation of the T2DM risk using the FINDRISC algorithm, which may have led to an underestimation of number of people with an increased risk for T2DM.

## 5 CONCLUSIONS

In this population‐based, cross‐sectional study, we demonstrate that the prevalence of hypertension, obesity, abdominal obesity and low HDL are common CVD risk factors in a predominately rural setting in Uganda. In addition, we show that HIV status has a limited impact on these results. The high prevalence of dyslipidaemia, particularly low HDL, is an important area for further study, but validated lipid abnormality cut‐offs in SSA need to be established. Finally, the non‐lab‐based Framingham 10‐year CVD risk score should be further explored for use for within rural or resource‐constrained settings considering limited CVD outcome data for the development of region‐specific CVD risk scores; while anthropometric measurements offer an area in which CVDs may be both prevented and captured early for clinical intervention.

## COMPETING INTERESTS

The authors have declared no competing interests.

## AUTHORS’ CONTRIBUTIONS

RE, RS, AN, BG, GM, FN, AME and HN contributed to the conception and design of the study. RE, GM, AME and HN contributed to the analysis and initial interpretation of the data for the study. RE, RS, AN GM, BG, LWC, SJR, FN, AME, NKS, DMS and HN contributed to the draft of the manuscript or provided substantial inputs, critical comments and suggested additional analyses. RE and HN finalized the manuscript. All authors read and approved the final manuscript.

## Supporting information


**Appendix Table A**: Cohort Characteristics by Sex and HIV Status for all participants aged 35–49.
**Appendix Table B**: Factors Associated with Total Cholesterol and High LDL Continued.

## Data Availability

None.
