# Supplementary material for: Prevalence of cardiovascular risk factors by HIV status in a population‐based cohort in South Central Uganda: a cross‐sectional survey
Source: J Int AIDS Soc. 2022 Apr 13;25(4):e25901. doi: 10.1002/jia2.25901 (PMC9008150; doi:10.1002/jia2.25901)
Supplement: Supplementary file 1 — Appendix Table A: Cohort Characteristics by Sex and HIV Status for all participants aged 35–49. Appendix Table B: Factors Associated with Total Cholesterol and High LDL Continued. [file JIA2-25-e25901-s001.docx]

| **Table A: Cohort Characteristics by Sex and HIV Status for all participants aged 35-49** | | | | | | |
| --- | --- | --- | --- | --- | --- | --- |
|  | **Females** | | **Males** | | **Total** | |
| **Characteristic** | **HIV+** No. (%) | **HIV-** No. (%) | **HIV+** No. (%) | **HIV-** No. (%) | **HIV+** No. (%) | **HIV-** No. (%) |
| **Age** | n=653 | n=2023 | n=362 | n=1827 | n=1015 | n=3850 |
| Age, mean (SD in years) | (41) | (41) | (41) | (41) | (41) | (41) |
| 35-39 | 267(41%) | 914 (45%) | 135 (37%) | 701 (38%) | 402 (40%) | 1615 (42%) |
| 40-44 | 243 (37%) | 697 (35%) | 130 (36%) | 654 (36%) | 373 (36%) | 1351 (35%) |
| 45-49 | 143 (22%) | 412 (20%) | 97 (27%) | 472 (26%) | 240 (24%) | 884 (23%) |
| **Residence Location** | n=653 | n=2023 | n=362 | n=1827 | n=1015 | n=3850 |
| Rural | 325 (50%) | 1132 (56%) | 197 (54%) | 1040 (57%) | 522 (51%) | 2172 (56%) |
| Semi-urban | 328 (50%) | 891 (44%) | 165 (46%) | 787 (43%) | 493 (49%) | 1678 (44%) |
| **Education** | n=653 | n=2021 | n=362 | n= 1827 | n=1015 | n=3848 |
| No Education | 50 (8%) | 123 (6%) | 17 (5%) | 74 (4%) | 67 (7%) | 197 (5%) |
| <5 Years of Study | 434 (66%) | 1523 (75%) | 261 (72%) | 1383 (76%) | 695 (68%) | 2906 (76%) |
| ≥5 Years of Study | 169 (26%) | 375 (19%) | 84 (23%) | 370 (20%) | 253 (25%) | 745 (19%) |
| **Occupation**^†^ | n=653 | n=2023 | n=362 | n=1827 | n=1015 | n=3850 |
| Agricultural Focused | 394 (60%) | 1300 (64%) | 166 (46%) | 864 (47%) | 560 (55%) | 2164 (56%) |
| Non-Agricultural Focused | 259 (40%) | 723 (36%) | 194 (54%) | 963 (53%) | 453 (45%) | 1686 (44%) |
| **Marital Status** | n=653 | n=2023 | n=362 | n=1827 | n=1015 | n=3850 |
| Single | 361 (55%) | 647 (32%) | 96 (27%) | 335 (18%) | 457 (45%) | 982 (26%) |
| Married | 292 (45%) | 1376 (68%) | 266 (73%) | 1492 (82%) | 558 (55%) | 2868 (74%) |
| **Religion** | n=653 | n=2022 | n=362 | n=1827 | n=1015 | n=3849 |
| None | 2 (<1%) | 9 (<1%) | 0 (0%) | 6 (<1%) | 2 (<1%) | 16 (<1%) |
| Catholic | 448 (69%) | 1319 (65%) | 259 (72%) | 1182 (65%) | 707 (69%) | 2501 (65%) |
| Protestant | 140 (21%) | 449 (22%) | 73 (20%) | 411 (23%) | 213 (21%) | 860 (22%) |
| Muslim | 63 (10%) | 245 (12%) | 30 (8%) | 227 (12%) | 93 (9%) | 472 (12%) |
| **Alcohol Consumption** | n=651 | n=2023 | n= 362 | n=1825 | n=1013 | n=3848 |
| No | 411 (63%) | 1140 (56%) | 131 (36%) | 609 (33%) | 542 (54%) | 1749 (45%) |
| Infrequent (last drink >1-month) | 41 (6%) | 195 (10%) | 27 (7%) | 87 (5%) | 68 (7%) | 282 (7%) |
| Frequent (last drink ≤1-month) | 199 (31%) | 688 (34%) | 204 (%) | 1129 (62%) | 403 (542%) | 1817 (47%) |
| **Smoking Status** | n=653 | n=2023 | n=362 | n=1827 | n=1015 | n=3850 |
| Non-smoker | 635 (97%) | 1950 (96%) | 294 (81%) | 1492 (82%) | 929 (92%) | 3442 (89%) |
| Smoker | 18 (3%) | 73 (4%) | 68 (19%) | 335 (18%) | 86 (8%) | 408 (11%) |
| **Duration of Smoking** | n=18 | n=73 | n=68 | n=335 | n=86 | n=408 |
| 1-10 Years | 11 (61%) | 59 (81%) | 34 (51%) | 164 (49%) | 45 (53%) | 223 (54%) |
| 11-20 Years | 6 (33%) | 10 (14%) | 25 (36%) | 101 (30%) | 31 (36%) | 111 (27%) |
| 21+ Years | 1 (6%) | 4 (5%) | 9 (13%) | 70 (21%) | 10 (11%) | 74 (18%) |
| **Physical Activity** | n=652 | n=2022 | n=361 | n=1826 | n=1013 | n=3848 |
| Physically Active (>30 minutes) | 640 (97%) | 1959 (97%) | 344 (95%) | 1767 (97%) | 974 (96%) | 3726 (97%) |
| Physically Inactive (≤30 minutes) | 22 (3%) | 63 (3%) | 17 (5%) | 59 (3%) | 39 (4%) | 122 (3%) |
| **Daily Fruit and Vegetable Consumption** | n=653 | n=2023 | n=362 | n=1827 | n=1015 | n=3850 |
| Consumption, median servings | 1.7 | 1.4 | 1.3 | 1.2 | 1.5 | 1.3 |
| Below average (<1.14 servings) | 218 (33%) | 862 (43%) | 203 (56%) | 1096 (60%) | 421 (41%) | 1958 (51%) |
| Above average (≥1.14 servings) | 435 (67%) | 1161 (57%) | 159 (44%) | 731 (40%) | 594 (59%) | 1892 (49%) |

SD = standard deviation

^†^Revised total for R18 cohort population aged 35-49 in the 37 communities of the RCCS are 1015 for HIV+, 3850 for HIV- and 14 for unknown HIV status. Differences from the initial reported total numbers (2 for HIV+, 3 for HIV- and 4 for unknown HIV status) are attributed to additional data cleaning related to participant ages and HIV status.

^‡^Information on those that reported to be unemployed (2 HIV+ males) not included in the table but included in the denominator shown.

| **Table B Factors Associated with Total Cholesterol and High LDL Continued** | | | | |
| --- | --- | --- | --- | --- |
|  | **Total Cholesterol** | | **High LDL** | |
|  | **Univariable**  **OR (95% CI)** | **Multivariable**  **OR (95% CI)** | **Univariable**  **OR (95% CI)** | **Multivariable**  **OR (95% CI)** |
| **HIV Status** |  |  |  |  |
| HIV- | Ref. | Ref. | Ref. | Ref. |
| HIV+ | 0.94 (0.72-1.23) | 1.02 (0.77-1.36) | 0.76 (0.59-0.99) | 0.85 (0.65-1.11) |
| **Sex** |  |  |  |  |
| Female | Ref. |  | **Ref.** | Ref. |
| Male | 0.98 (0.74-1.30) | – | **0.69 (0.52-0.91)** | 1.04 (0.73-1.46) |
| **Age** |  | – |  |  |
| 35-39 | **Ref.** |  | Ref. | – |
| 40-44 | 1.00 (0.72-1.37) | – | 0.97 (0.72-1.30) | – |
| 45-49 | 1.28 (0.91-1.80) | – | 1.10 (0.80-1.52) | – |
| **Residence Location** |  |  |  |  |
| Rural | **Ref.** | Ref. | **Ref.** | Ref. |
| Semi-Urban | **1.63 (1.24-2.14)** | 1.24 (0.93-1.66) | **1.61 (1.25-2.09)** | 1.28 (0.97-1.68) |
| **Education** |  |  |  |  |
| No education | 0.55 (0.27-1.11) | – | **0.30 (0.13-0.68)** | **0.36 (0.15-0.83)**** |
| <5 years of study | 0.83 (0.60-1.17) | – | **0.77 (0.56-1.06)** | 0.89 (0.64-1.24) |
| ≥5 years of study | Ref. | – | **Ref.** | Ref. |
| **Occupation** |  |  |  |  |
| Agricultural Focused | **0.67 (0.58-0.77)** | **0.72 (0.62-0.83)****** | **0.76 (0.67-0.86)** | **0.83 (0.73-0.96)**** |
| Non-Agricultural Focused | **Ref.** | Ref. | **Ref.** | **Ref.** |
| **Smoking Status** |  |  |  |  |
| Non-smoker | Ref. | – | **Ref.** | Ref. |
| Current Smoker | 1.04 (0.66-1.65) | – | **0.65 (0.39-1.08)** | 0.86 (0.50-1.47) |
| **Physical Activity** |  |  |  |  |
| Physically Active (>30 minutes) | Ref. | – | Ref. | – |
| Physically Inactive (≤30 minutes) | 0.86 (0.39-1.91) | – | 0.99 (0.48-2.02) | – |
| **Daily Fruit and Vegetable Consumption** |  |  |  |  |
| Below average (<1.14 servings) | 0.97 (0.74-1.28) | – | 0.96 (0.75-1.24) | (-) |
| Above average (≥1.14 servings) | Ref. | – | Ref. | Ref. |
| **BMI** |  |  |  |  |
| Underweight (<18.5 kg/m^2^) | **0.90 (0.48-1.68)** | 0.96 (0.51-1.80) | **0.97 (0.54-1.73)** | 1.08 (0.60-1.95) |
| Normal (≥18.5 to <25 kg/m^2^) | **Ref.** | Ref. | **Ref.** | Ref. |
| Overweight (≥25 to <30 kg/m^2^) | **2.20 (1.59-3.03)** | 1.44 (0.96-2.17) | **2.75 (2.04-3.71)** | **1.69 (1.15-2.48)***** |
| Obese (≥30 kg/m^2^) | **3.48 (2.35-5.14)** | 1.67 (0.93-3.02) | **3.45 (2.36-5.05)** | 1.25 (0.71-2.20) |
| **Waist Circumference** |  |  |  |  |
| Not at risk (males: <95 cm & females: <81 cm) | **Ref.** | Ref. | **Ref.** | Ref. |
| Increased risk (males: ≥95 to ≤102 cm & females: ≥81 to ≤88 cm) | **1.62 (1.11-2.35)** | 1.27 (0.82-1.96) | **1.54 (1.07-2.20)** | 1.17 (0.75-1.82) |
| Substantially increased risk (males: ≥103 cm & females: ≥89 cm) | **3.48 (2.52-4.79)** | **1.95 (1.13-3.35)**** | **4.11 (3.04-5.56)** | **2.58 (1.51-4.43)***** |
| **Waist-to-hip Ratio** |  |  |  |  |
| Not at risk (males: <0.90 & females: <0.85) | **Ref.** | Ref. | **Ref.** | Ref. |
| Substantially increased risk (males: ≥0.90 & females: ≥0.85) | **1.73 (1.32-2.28)** | 1.19 (0.86-1.63) | **1.76 (1.36-2.27)** | **1.14 (0.84-1.54)** |

BMI=body mass index.

Significance p-levels: *p <.20, **p <.05, ***p <.01, ****p <.001
